# Early-life exposures, biological age acceleration, and type 2 diabetes in adulthood: mediation analyses in the UK Biobank

**DOI:** 10.1007/s11357-026-02138-6

**Published:** 2026-02-07

**Authors:** Denghui Hu, Diana van Heemst, Qiuyu Feng, Ko Willems van Dijk, Raymond Noordam

**Affiliations:** 1 Department of Human Genetics, Leiden University Medical Center, Leiden, the Netherlands; 2 Department of Internal Medicine, Section of Gerontology and Geriatrics, Leiden University Medical Center, Leiden, the Netherlands; 3 Department of Internal Medicine, Division of Endocrinology, Leiden University Medical Center, Leiden, the Netherlands; 4 Department of Clinical Epidemiology, Leiden University Medical Center, Leiden, the Netherlands; 5 Health Campus the Hague/Public Health and Primary Care, Leiden University Medical Center, the Hague, the Netherlands

**Keywords:** Early-life exposures, Biological age acceleration, Mediation, Type 2 diabetes

## Abstract

Early-life exposures could affect the risk of type 2 diabetes (T2D) in adulthood, but the mechanisms remain unclear. We included 319,951 (54.5% women) UK Biobank participants free of T2D at baseline and within the first year of follow-up to investigate whether early-life exposures contribute to T2D through biological aging estimated using the Klemera–Doubal (KDM) and PhenoAge methods. We first examined associations of exposures with biological age and T2D, respectively, using multivariable-adjusted linear models and Cox regression models. Next, we investigated the role of biological age acceleration in exposure-T2D associations using mediation analyses. Overall, the mean (standard deviation) chronological age of participants was 56.3 (8.1) years, KDM age was 40.9 (13.1) years, and PhenoAge was 44.4 (10.0) years. 17,062 T2D cases developed during a median (interquartile range) follow-up of 14.3 (13.5–15.0) years. Maternal smoking around birth, being part of a multiple birth, earlier puberty, and being relatively plumper or thinner at age ten were associated with both a higher biological age and T2D risk, while having a higher birth weight and being breastfed were associated with a lower T2D risk. Biological age acceleration partly mediated the exposure-T2D associations, ranging from a proportion of 8.3% (95%CI: 6.0–13.1%) of the association between birthweight-T2D being mediated by PhenoAge to a proportion of 27.7% (95%CI: 15.3–48.5%) of the association between breastfeeding-T2D being mediated by KDM. In conclusion, early-life exposures were associated with biological age acceleration that partly mediated the exposure-T2D associations, highlighting the importance of addressing early-life risks and biological aging in prevention strategies.

**Supplementary Information:**

The online version contains supplementary material available at https://doi.org/10.1007/s11357-026-02138-6.

## Introduction

It is increasingly recognized that risk exposures during the periconceptional, fetal, and early childhood stages of life are associated with an increased long-term risk of metabolic disorders, in line with the *Developmental Origins of Health and Disease* (DOHaD) framework [1]. For example, previous studies have associated lower birth weight and unhealthy infant feeding practices with an increased risk of Type 2 diabetes (T2D) later in life [2–4]. Although the underlying mechanisms remain poorly understood, the DOHaD framework hypothesizes that changes in the regulation of energy balance when exposed to these early risks may result in adaptive programs that, while beneficial early in life, may disrupt systemic homeostasis and increase the risk of disease later in life, especially when mismatches occur with adult life exposures [1, 5, 6].

In contrast, although not mutually exclusive, evolutionary theories of aging proposed that early-life exposures may contribute to an accelerated general biological decline with aging, due to a higher rate of damage accumulation on molecular, cellular, and organic/systemic levels [7, 8]. In this context, biological aging, a concept distinct from chronological aging, reflects the cumulative functional impact of exposures on a biological level, which can be assessed by analyzing genetic, epigenetic, metabolic, proteomic, or clinical biomarkers, thereby providing tools to quantitatively measure the long-term effects of exposures [9, 10]. Using such an approach, a previous study observed that individuals exposed to prenatal famine had an accelerated biological age, as measured by epigenetic clocks, even sixty years later [11].

In recent studies, biological aging is also increasingly recognized as a shared disease pathway and key predictor of age-related outcomes, including cardiometabolic diseases and mortality [12, 13]. T2D is a typical aging-related disease, whose incidence strongly rises with increasing age, accompanied by cumulative biological deterioration across multiple systems, including muscle, adipose and liver tissue. While traditional risk factors (e.g., age, body mass index, and sex) explain around 50–60% of the risk of T2D [14, 15], these do not fully account for the current and projected risks of T2D. A previous study confirmed that early-onset T2D is associated with multiple perinatal factors [2]. In the context of these observations and taking into account the theories of biological aging, linking early-life exposures to T2D risk through biological age acceleration may help further understand the pathways of disease development from a life-course perspective. Ultimately, this may contribute to bridging the evidence gap and informing future prevention strategies.

**Fig. 1:**
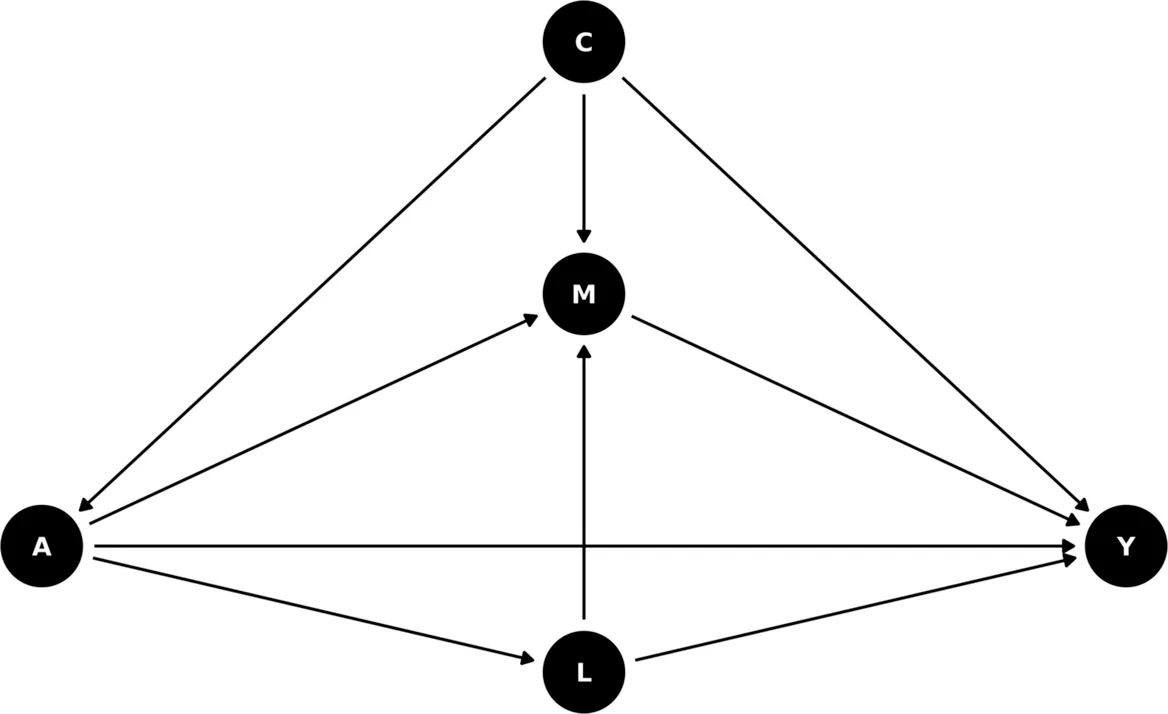
Directed acyclic graph depicting potential associations between early-life exposures, biological ageing and type 2 diabetes. A, exposures (early-life risks); M, mediators (biological age acceleration); Y, outcome (type 2 diabetes); C, baseline confounders not affected by the exposure (e.g., sex, ethnicity); L, post-exposure confounders affected by the exposure (e.g., body mass index, health behaviors)

To the best of our knowledge, it remains unclear whether and to what extent associations between early-life exposures and late-life T2D risk are mediated by biological age acceleration (BAA). We therefore aimed to investigate the following hypotheses as shown in the directed acyclic graph (DAG) framework (Fig. 1), using participants from the UK Biobank (UKB): (i) early-life exposures are associated with the risk of incident T2D; (ii) early-life exposures are associated with BAA; (iii) higher BAA is associated with a higher risk of incident T2D; and (iv) BAA mediates, at least in part, the associations between early-life exposures and T2D.

## Methods

### Study setting and population

This study used data from the UKB, which recruited over 500,000 participants aged 40–73 years between 2006 and 2010 across the entire UK, and participants had a completed assessment of the physical, biological, and socioeconomical status. To date, longitudinal follow-up for a wide range of health outcomes is still ongoing for all participants. Recruitment and follow-up procedures were approved by the North West Multi-Centre Research Ethics Committee (MREC) and complied with UK ethical and regulatory standards [16]. All participants provided written informed consent via electronic signature. The study adhered to the principles of the Declaration of Helsinki and data usage following relevant UK governance frameworks (application number 56340).

From the original cohort of 502,393 participants, this study excluded 182,443 participants who lacked data on blood biochemical/clinical indicators, had extreme biomarker values that exceeded five standard deviations (SD), or had a history of T2D at baseline or were diagnosed with T2D within one year after recruitment. These exclusions resulted in a final sample size of 319,951 participants available for analyses (Supplementary Figure S1). For each analysis, we further excluded participants with missing exposure and covariate data. The final sample size varied by analysis and is reported alongside the results in the corresponding tables or figures.

### Early life exposures

At recruitment, UKB participants completed questionnaires via touchscreen/verbal interview to provide information on early-life factors. Data on exposure to maternal smoking around birth (UKB Field 1787), being part of a multiple birth (UKB Field 1777), birth weight (UKB Field 20,022), being breastfed as a baby (UKB Field 1677), body size (UKB Field 1687) and height (UKB Field 1697) at age ten, age of menarche for women (UKB Field 2714), and relative age at first facial hair for men (UKB Field 2375) were obtained through self-reported questions. Values of birth weight were converted to kilograms (kg) by the UKB team. Birth weight was analyzed both as a continuous variable and as categories: low (< 2.5 kg), normal (2.5–3.5 kg), middle to high (3.5–4.5 kg), and high (> 4.5 kg). Childhood body size and height were classified into three groups (thinner/shorter, average, and plumper/taller). Age of menarche was categorized as younger (< 12 years), average (12–13 years), and older (> 13 years). The relative puberty age was derived by combining both sex-specific indicators, the age of menarche and the relative age at first facial hair. Other exposures were coded as binary variables (yes/no). For each variable, participants who reported “Do not know” or “Prefer not to answer” were labelled with missing values and excluded from the analyses.

### Ascertainment of T2D

The occurrence of T2D (E11) diagnosis was defined using the three-character International Classification of Diseases, Tenth Revision (ICD-10) codes, based on linkage data of the primary care records, hospital inpatient records, death records, and self-reported diagnoses. The outcome dates of T2D diagnoses/codes (UKB Field 130,708) were defined as the first recorded across all the above data sources, generated by a standard algorithm from the UKB team (https://biobank.ndph.ox.ac.uk/showcase/exinfo.cgi?src=Data_providers_and_dates). Follow-up for individuals extended from baseline (2006–2010) until the first registration of a diagnosis code of T2D, loss to follow-up, death, or the end of the study period (July 31, 2023), whichever came first.

### Biological age acceleration

There is no gold standard for estimating biological age. However, the Klemera–Doubal method (KDM) and PhenoAge method, both based on routinely-measured physiological and laboratory biomarkers, are the best validated, repeatable, cost-effective, and non-invasive approaches currently available [10], and therefore, chosen to be used in the present study. We applied the KDM and PhenoAge algorithms based on the National Health and Nutrition Examination Survey (NHANES) (Overview in Supplementary Figure S1), a nationally representative dataset with standardized biomarker measurements and long-term follow-up, for model training and testing, and subsequently projected onto the UKB study population.

We adapted the original KDM and PhenoAge algorithms to the 12 biomarkers consistently available in both NHANES and UK Biobank, guided by the original algorithm specifications and informed by previous studies [9, 12]. For the KDM biological age, we used 8 biomarkers, including C-reactive protein (CRP), forced expiratory volume, glycated hemoglobin, creatinine, alkaline phosphatase, albumin, total cholesterol, and systolic blood pressure. PhenoAge was estimated from 10 biomarkers (CRP, white blood cell count, mean corpuscular volume, red cell distribution width, lymphocyte percentage, glycated hemoglobin, creatinine, alkaline phosphatase, albumin, and total cholesterol). Details of biomarkers’ collection and UKB field ID are reported in the Supplementary Table S1-S2. KDM estimates biological age as the chronological age at which an individual’s physiological profile according to the biomarkers mentioned above would be normal in a reference population from n regression lines, whereas PhenoAge estimates biological age as the chronological age corresponding to a normal mortality risk from elastic net Gompertz regression [9]. Despite being derived from different outcomes and algorithms, these two biological age measures often yield consistent results, reinforcing their utility across aging-related research [12, 17]. The biological ages were estimated using the ‘BioAge’ package in R, following the steps:

First, we trained our KDM (stratified by sex) and PhenoAge algorithms separately in the third NHANES dataset (1988–1994). Subsequently, we projected the models onto NHANES IV (1999–2018) dataset for new algorithm testing. We compared our modified KDM age and PhenoAge with the results from the original KDM and PhenoAge algorithm with original biomarkers by calculating the Pearson correlation with chronological age. Both our modified KDM and PhenoAge exhibited a strong correlation with chronological age, with correlation coefficients of 0.60 and 0.96 in NHANES IV and correlations of 0.85 and 0.61 in UKB (Supplementary Figure S2-S3). We also checked the associations of our modified biological ages with mortality in NHANES IV (Supplementary Table S3). We subsequently projected the algorithms onto the data from UKB participants and calculated their biological ages with a sample size of 319,951 participants. BAA was defined as the residual from regressing biological age on chronological age, and standardized to a mean of zero and a SD of one for comparability across analyses. To evaluate robustness, we implemented a leave-one-out biomarker strategy, which demonstrated stable estimations of biological ages in UKB with correlations between each leave-one-out biological age and the global biological age. Except for KDM when excluding CRP (r^2^ = 0.65), all correlation coefficients exceeded 0.80, with the majority above 0.95 (Supplementary Figures S4–S5).

### Covariates

According to the DAG framework (Fig. 1), we identified two main types of confounders. First, baseline factors that confound the associations of early-life exposures with both biological age accelerations and type 2 diabetes, including sex, age, ethnicity (White/other), polygenic risk scores (PRS) for T2D, and family history of T2D. Second, exposure-induced mediator–outcome confounders (post-exposure confounders) that could be influenced by early-life exposures, including body mass index (BMI), education level (high, medium, low, or unknown), Townsend deprivation index at baseline, smoking status, drinking status, and use of cholesterol- or blood pressure-lowering medications. Data were collected at baseline with self-reported questionnaires from a touchscreen/verbal interview. PRS was derived from UKB genotype data using published T2D GWAS variants, followed by standard quality control and weighted allele summation [18].

### Statistical analysis

Continuous variables were summarized as mean (SD) and/or Median (interquartile range, IQR) depending on their distribution, and categorical variables as percentages. We first examined the associations between early-life exposures and T2D incidence. Then, only those exposures associated with T2D were further examined in relation to BAA. Multivariable-adjusted linear regression models were fitted between each exposure and BAA, while Cox proportional hazards models were applied between exposures and T2D, as well as between BAA and T2D. Results were reported as coefficients or hazard ratios (HR) with 95% confidence intervals (CI) and visualized using heatmaps and forest plots to summarize the overall result patterns. Then, we conducted mediation analyses using the g-formula [19] for T2D-associated exposures and BAA. This method decomposes the total effect of the exposure on T2D into a randomized direct effect operating independently of BAA and a randomized indirect effect mediated through BAA, while appropriately accounting for post-exposure confounders by modelling the sequential data-generating process of confounders, mediator, and outcome. The proportion mediated (PM) was calculated, representing the fraction of the exposure–outcome association explained by BAA. Interaction terms between exposures and mediators were also considered. Statistical inference was based on bootstrap resamples to derive 95% CIs, ensuring robust and reproducible estimates. We performed bootstrap resampling with 500 and 1000 replicates to evaluate the stability of confidence intervals (Supplementary Table S4-S5). Sensitivity analyses using the E-value were performed to assess the potential impact of unmeasured confounding [20]. All analyses were conducted in R (version 4.4.0). Mediation analyses were performed with the ‘CMAverse’ package in R.

## Results

### Baseline characteristics of participants

The study characteristics of the exposures, KDM, and PhenoAge, stratified by sex, are summarized in Table 1. A total of 319,951 T2D-free participants (54.5% women) had a mean (SD) baseline chronological age of 56.3 (8.10) years. Although women and men had a similar chronological mean age, women had a higher mean KDM biological age (47.4 ± 8.59 years) compared to men (33.0 ± 13.3 years), while their mean PhenoAge was similar (43.5 ± 9.69 years in women *versus* 45.4 ± 0.96 years in men). Median KDM age and PhenoAge are also reported in Supplementary Table S6.

**Table 1:** Characteristics of the study population stratified by sex. *N*(%)

	Female (*N***=** 174,532)	Male (*N*** = **145,419)	Total (*N*** = **319,951)
Chronological age, years	56.2 (7.99)	56.4 (8.23)	56.3 (8.10)
Biological age, years			
KDM: Mean (SD)	47.4 (8.59)	33.0 (13.3)	40.9 (13.1)
KDM acceleration Z-scaled: Mean (SD)	0.64 (0.32)	−0.77 (1.00)	0 (1.00)
PhenoAge: Mean (SD)	43.5 (9.69)	45.4 (10.20)	44.4 (9.98)
PhenoAge acceleration Z-scaled: Mean (SD)	−0.15 (1.01)	0.18 (0.96)	0 (1.00)
Follow-up years: Median (IQR)	14.4 [13.6, 15.0]	14.3 [13.4, 15.0]	14.3 [13.5, 15.0]
Being part of a multiple birth			
No	167,797 (97.7)	139,737 (97.8)	307,534 (97.7)
Yes	3961 (2.3)	3149 (2.2)	7110 (2.3)
Missing ^a^	2774 (1.6)	2533 (1.7)	5307 (1.7)
Birthweight, kg			
Mean (SD)	3.24 (0.63)	3.45 (0.68)	3.32 (0.66)
Low	62,121 (56.7)	33,553 (47.9)	95,674 (53.3)
Normal	12,632 (11.5)	5064 (7.2)	17,696 (9.9)
Middle to High	31,240 (28.5)	26,083 (37.3)	57,323 (31.9)
High	3569 (3.3)	5294 (7.6)	8863 (4.9)
Missing	64,970 (37.2)	75,425 (51.9)	140,395 (43.9)
Breastfed as a baby			
No	42,124 (29.8)	25,665 (24.6)	67,789 (27.9)
Yes	99,200 (70.2)	78,769 (75.4)	177,969 (72.4)
Missing	33,208 (19.0)	40,985 (28.2)	74,193 (23.2)
Maternal smoking			
No	108,540 (71.6)	87,355 (70.1)	195,895 (71.0)
Yes	42,948 (28.4)	37,207 (29.9)	80,155 (29.0)
Missing	23,044 (13.2)	20,857 (14.3)	43,901 (13.7)
Puberty age			
Younger	33,378 (19.7)	9438 (6.7)	42,816 (13.9)
Average	73,682 (43.6)	112,036 (80.1)	185,718 (60.1)
Older	62,049 (36.7)	18,409 (13.2)	80,458 (26.0)
Missing	5423 (3.1)	5536 (3.8)	10,959 (3.4)
Body size at age 10			
Average	87,711 (51.1)	73,575 (51.8)	161,286 (51.4)
Thinner	54,084 (31.5)	49,608 (34.9)	103,692 (33.1)
Plumper	29,740 (17.3)	18,813 (13.2)	48,553 (15.5)
Missing	2997 (1.7)	3423 (2.4)	6420 (2.0)
Height size at age 10			
Average	9,1516 (53.5)	79,227 (55.4)	170,743 (54.4)
Shorter	35,999 (21.1)	27,886 (19.5)	63,885 (20.4)
Taller	43,450 (25.4)	35,805 (25.1)	79,255 (25.2)
Missing	3567 (2.0)	2501 (1.7)	6068 (1.9)

a Percentages for each category are calculated using the number of non-missing observations as the denominator. Missing values are reported separately and excluded from each analysis. *T2D *type 2 diabetes, *SD* standard deviation, *IQR* interquartile range, *KDM* Klemera–Doubal method biological age,* PhenoAge* Levine method biological age, *kg* kilogram

Only 2.3% of participants were part of a multiple birth. The overall mean birth weight of the total participants was 3.32 (0.66) kg. Women had a lower birth weight than men, as shown both in the mean birth weight (3.24 ± 0.63 kg in women *versus* 3.45 ± 0.68 kg in men) and proportions in Table 1. About three-quarters of the study population (72.4%) had been breastfed as a baby, and 29.0% were exposed to maternal smoking around birth. Higher proportions of male participants (80.1%) had their puberty in an average year than female participants (43.6%). Regarding body size and height at age ten, more than half of the participants reported being of average size. Additional baseline demographic and lifestyle characteristics, including socioeconomic indicators, BMI, smoking, and medication use, are provided in Supplementary Table S6.

### Associations of exposures and BAA with T2D events

**Fig. 2:**
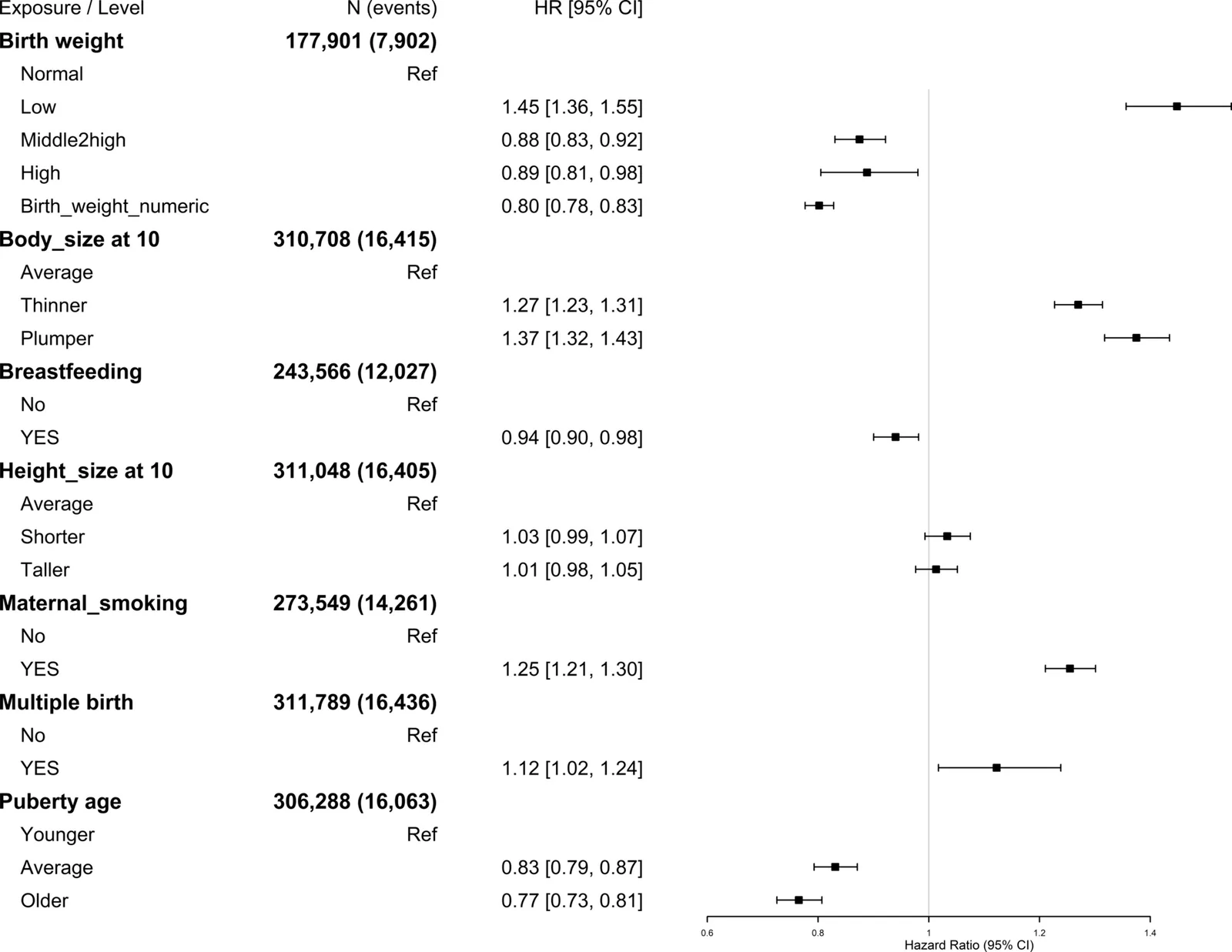
Forest plot of Cox associations among early-life exposures and T2D events. *N*, sample size for each model; *HR*, hazard ratio; *CI*, confidence interval; Ref, reference

Among 319,951 T2D-free participants, 17,062 developed T2D during a median (IQR) follow-up of 14.3 (13.5–15.0) years. Figure 2 presents the HRs from the multivariable-adjusted Cox regression analyses for the associations between early-life exposures and incident T2D. Different body heights at age ten years, both being taller and shorter, were not associated with incidence of T2D compared with the reference group with average height at age ten. Birth weight demonstrated a potential linear relationship with T2D risk that participants with low birth weight had a 1.45-fold higher risk (95% CI: 1.36–1.55), whereas those with middle-to-high and high birth weight had lower risks (HR = 0.88, 95% CI: 0.83–0.92; HR = 0.89, 95% CI: 0.81–0.98, respectively) compared with normal birth weight. The quantitative analysis confirmed that each unit increase in birth weight was associated with a lower T2D risk (HR = 0.80, 95% CI: 0.78–0.83).

Being thinner or plumper at age ten, compared to average body composition, was associated with an increased T2D risk (HR = 1.27, 95% CI: 1.23–1.31; HR = 1.37, 95% CI: 1.32–1.43, respectively). Being breastfed as a baby was associated with a lower risk of incident T2D (HR = 0.94, 95% CI: 0.90–0.98). In contrast, exposure to maternal smoking around birth (HR = 1.25, 95% CI: 1.21–1.30) and being part of a multiple birth (HR = 1.12, 95% CI: 1.02–1.24) were associated with higher risks for incident T2D. Relatively later puberty onset was associated with a lower risk of incident T2D, with HRs of 0.83 (95% CI: 0.79–0.87) for average puberty age and 0.77 (95% CI: 0.73–0.81) for relatively older puberty age compared with a relatively younger puberty age.

The associations between BAA and incident T2D are presented in Table 2. Both z-scored KDM and PhenoAge acceleration were positively associated with T2D risk, whether modelled per SD or by quartiles, showing dose–response relationships. Per SD increase, KDM acceleration was associated with a 1.52-fold higher risk of T2D (95% CI: 1.49–1.55), and PhenoAge acceleration with a 1.47-fold higher risk (95% CI: 1.45–1.49).

**Table 2:** Risk of T2D dependent on biological age acceleration

Model	Exposures	T2D risk (HR)	95% CI	
Model 1 ^a^	KDM-BAA per SD	1.52	1.49	1.55
Model 2 ^b^	KDM-BAA-quartile1	Reference		
	KDM-BAA-quartile2	1.56	1.48	1.63
	KDM-BAA-quartile3	1.77	1.66	1.88
	KDM-BAA-quartile4	2.92	2.77	3.09
Model 3 ^a^	PhenoAge-BAA per SD	1.47	1.45	1.49
Model 4 ^b^	PhenoAge-BAA-quartile-1	Reference		
	PhenoAge-BAA-quartile-2	1.53	1.44	1.64
	PhenoAge-BAA-quartile-3	2.04	1.92	2.17
	PhenoAge-BAA-quartile-4	3.23	3.05	3.43

a Model 1 and Model 3 included scaled continuous biological age accelerations per standard deviation as predictors. b, Model 2 and Model 4 included quartile biological age accelerations determined by media and quarter ranges as predictors. All models adjusted for age, sex, ethnicity, polygenetic risk scores, family histories, body mass index, educational groups, Townsend deprivation index, ever smoking, ever drinking, medication use for cholesterol, and medication use for blood pressure. *SD* standard deviation, *KDM-BAA *Klemera–Doubal method biological age acceleration,* PhenoAge-BAA* Levine method biological age acceleration, *HR* hazard ratio, *CI *confidence interval

### Associations between the early-life exposures and BAA

**Fig. 3:**
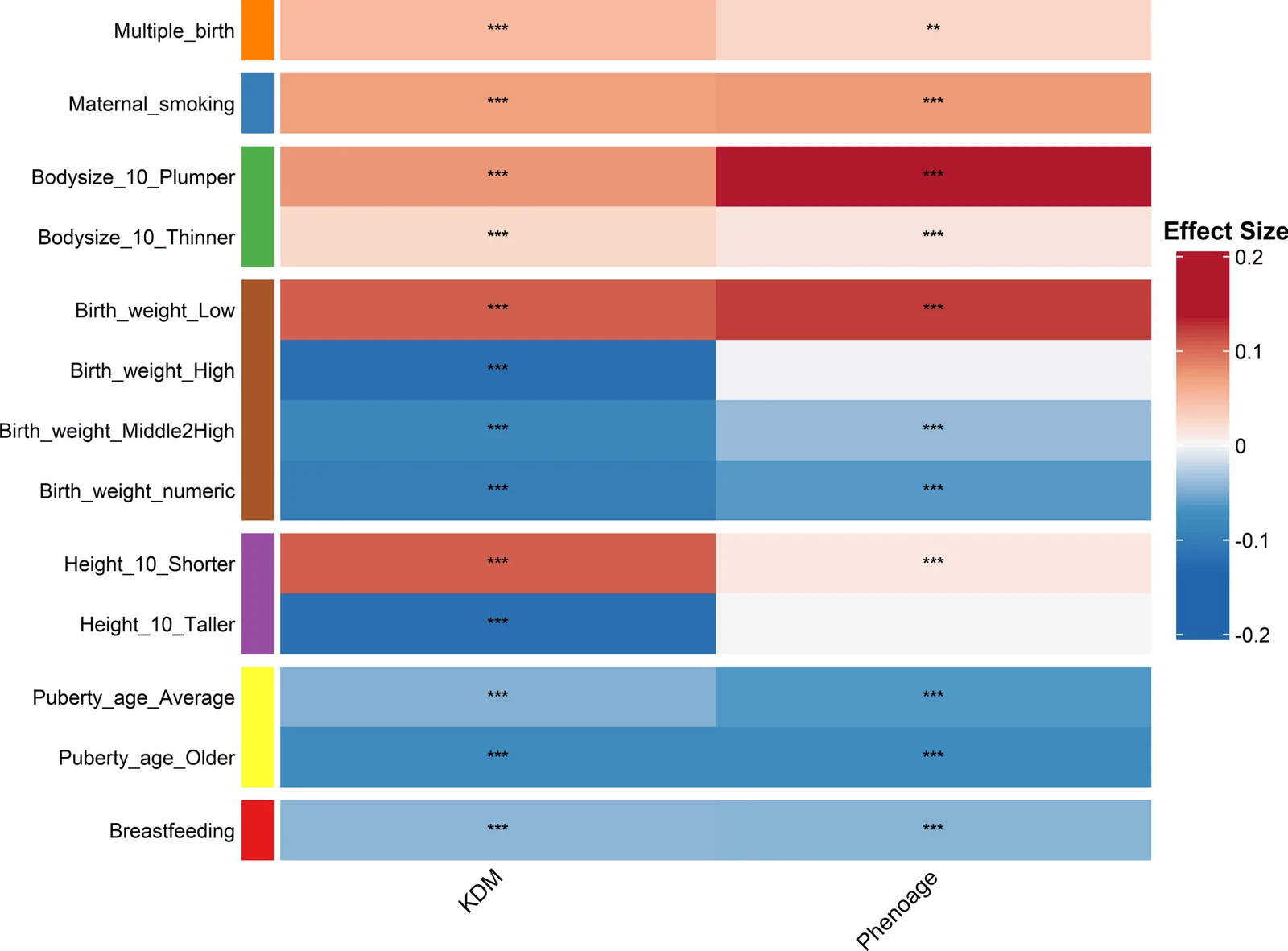
Heatmap of linear associations among KDM, PhenAge, and early-life exposures. * *P* value less than 0.05; ** *P* value less than 0.01; *** *P* value less than 0.001. *KDM*, Klemera–Doubal method biological age; *PhenoAge*, Levine method biological age

After adjusting for age, sex, ethnicity, PRS, and family history of T2D, early-life exposures were associated with KDM and PhenoAge acceleration, as shown in Fig. 3. Higher birth weight, being taller than average at age ten, later pubertal onset, and being breastfed were associated with lower KDM and PhenoAge acceleration, while being part of a multiple birth, exposed to maternal smoking around birth, and being plumper/thinner at age ten showed associations with higher KDM and PhenoAge acceleration. More specifically, breastfeeding was associated with 0.044 (95% CI: 0.038–0.051) and 0.046 (95% CI: 0.038–0.055) SD lower KDM and PhenoAge acceleration, respectively. Exposure to maternal smoking around birth was associated with higher KDM and PhenoAge acceleration, with 0.069 (95% CI: 0.063–0.075) SD and 0.074 (95% CI: 0.066–0.082) SD, respectively. Additionally, a dose–response relationship was observed between birth weight and KDM acceleration. Compared with normal birth weight, middle-to-high birth weight was associated with a 0.083 (95% CI: 0.076–0.090) SD decrease in KDM BAA, and high birth weight with a 0.116 (95% CI: 0.102–0.131) SD decrease.

### Mediation of biological age acceleration

**Fig. 4:**
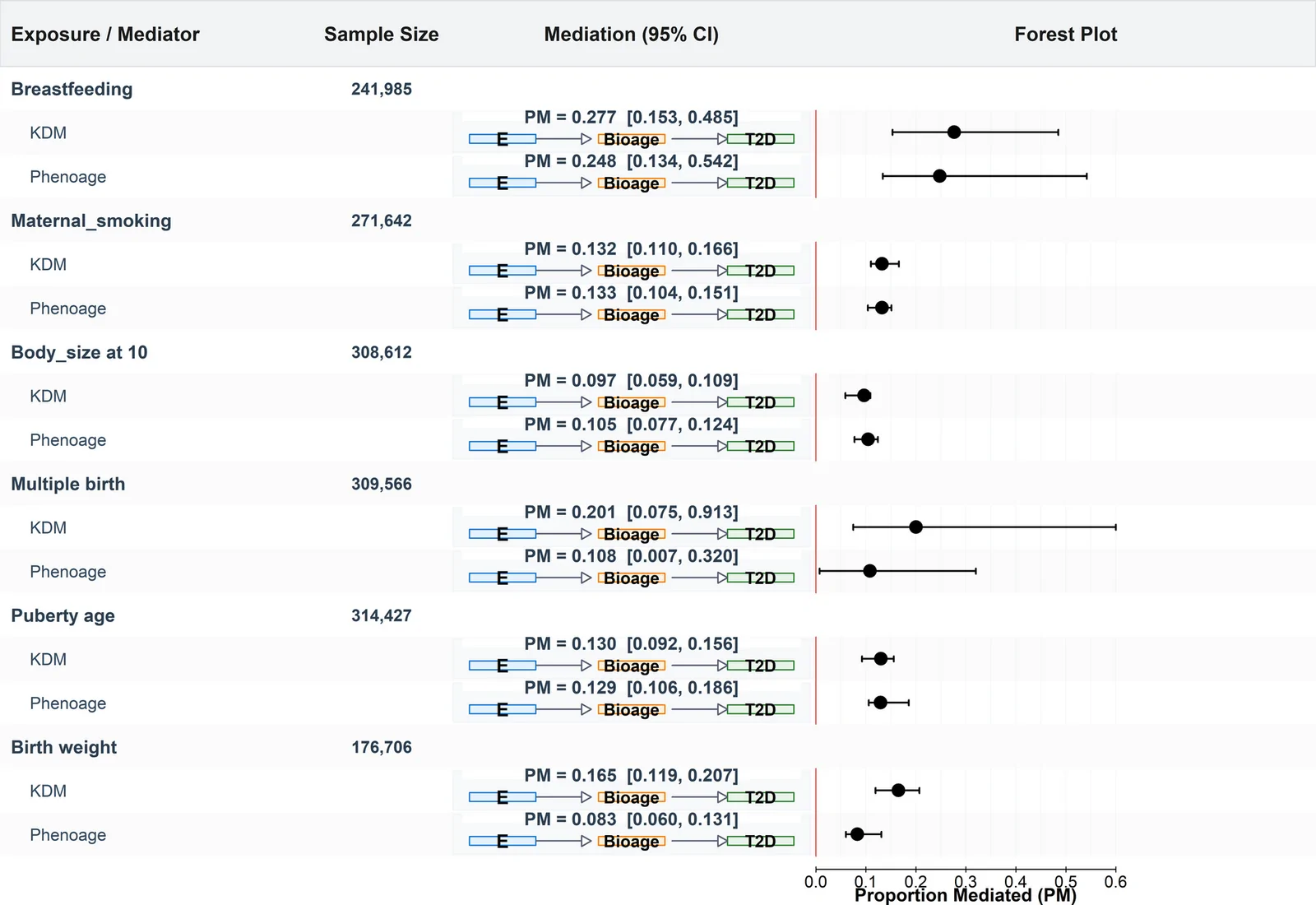
Forest plot of mediated proportions of KDM and PhenoAge biological age acceleration in the relationships among early-life exposures and T2D events estimated by bootstrapping. *KDM*, Klemera–Doubal method biological age;* PhenoAge*, Levine method biological age; *PM*, proportion mediated

Figure 4 presents the mediation estimates of KDM and PhenoAge acceleration in the association between early-life exposure and incident T2D. For being breastfed as a baby, KDM explained 27.7% (95% CI: 15.3%−48.5%) of the protective association with T2D, while PhenoAge accounted for 24.8% (95% CI: 13.4%−54.2%). In the case of maternal smoking around birth, KDM and PhenoAge explained 13.2% (95% CI: 11.0%−16.6%) and 13.3% (95% CI: 10.4%−15.1%) of the excess T2D risk, respectively. Body size at age ten showed smaller mediation effects, with 9.7% (95% CI: 5.9%−10.9%) explained by KDM and 10.5% (95% CI: 7.7%−12.4%) by PhenoAge.

For being part of a multiple birth, KDM accounted for 20.1% (95% CI: 7.5%−91.3%) of the association, whereas PhenoAge explained 10.8% (95% CI: 0.7%−32.0%) of the association. Regarding puberty age, 13.0% (95% CI: 9.2%−15.6%) of the protective effect was explained by KDM and 12.9% (95% CI: 10.6%−18.6%) by PhenoAge. Finally, for birth weight, KDM explained 16.5% (95% CI: 11.9%−20.7%) of the association, and PhenoAge explained 8.3% (95% CI: 6.0%−13.1%). We also identified several significant interactions among exposures and mediators, as shown in the Supplementary Table S7.

## Discussion

In this prospective analysis using UKB data, we assessed the associations between early-life exposures, biological age acceleration, and T2D incidence. We found protective associations for being breastfed as a baby and having a higher birthweight, and risk associations for being exposed to maternal smoking around birth, being part of a multiple birth, having an early puberty age, and a thinner/plumper body size at age ten. Biological age acceleration partly mediated these associations, with mediation proportions reaching up to 27.7%. The observed mediations were consistent across the two biological age measures (KDM and PhenoAge), both of which were derived from algorithms not reliant on a single biomarker. These findings further support the relationship between early-life risks, biological aging, and late-life T2D, highlighting the role of early-life risks in accelerated biological aging and then T2D development.

As recognized, and consistent with previous studies, we confirmed the previously observed associations between early-life exposures and T2D incidence [4, 21, 22]. Specifically, we observed that higher birth weight was associated with a lower incidence of T2D, in line with earlier findings [23]. Children with low birth weight may undergo catch-up growth, which can lead to disproportionate fat accumulation and insulin hyper-responsiveness [22]. Results of maternal smoking around birth and not being breastfed were also consistent with previous reports showing associations between these exposures and higher T2D incidence [21, 24]. We also observed similar findings that being part of a multiple birth, having an early puberty age, and having either a thinner or plumper body size at age ten were risk factors for T2D events [22, 25]. In addition to the well-established exposure-T2D associations, our study provides evidence that early-life exposures are associated with biological age acceleration in later life, as captured by KDM and PhenoAge, which is under-investigated and may support hypotheses regarding the long-term impact of early-life risks. For example, the evolutionary aging theory suggests that early-life exposures may trigger long-term cumulative damage [8]. This, in turn, can accelerate aging processes across multiple organ systems and contribute to the development of T2D [7].

To the best of our knowledge, there is only one prior UKB study that observed that breastfeeding, maternal smoking, and low birth weight were associated with clinical measures of biological age; however, it focused more on interactions of exposures with chronological age and late life environmental risks, and included a narrower set of early-life exposures [26]. Importantly, our study quantified that KDM and PhenoAge acceleration could mediate part of the associations between the specific early-life exposures during prenatal, postnatal, and childhood periods, and late-life T2D incidence. This further extends a previous study where KDM and PhenoAge mediated the association between a composite score of early-life risks (exposed to maternal smoking, not breastfed as baby, and lower birth weight) and the development of T2D in adulthood. Beyond this, to the best of our knowledge, only a few studies have examined the mediating role of BAA in the association between obesity and cardiovascular disease, further highlighting the novelty and potential significance of our observations [27]. Nevertheless, the mediation proportions for biological age acceleration were moderate (8% to 27.7%), indicating that accelerated biological aging explains only part of the association between early-life exposures and T2D, and that other biological and social pathways are likely to be involved.

Interestingly, our results suggest some potential interactions between early-life exposures and BAA in the associations with T2D, indicating that the joint influence of early-life risks and accelerated biological age may be more complex than simple mediation. Although exploratory, this finding points to the potential value of interventions that either reduce adverse early-life exposures or target biological aging-related lifestyles as potential strategies to mitigate later-life T2D risk. However, because aging reflects a multifactorial and dynamic integration of diverse biological pathways, disentangling these findings remains challenging and requires cautious interpretation [28]. This complexity highlights an alternative paradigm that changes in clinical biomarkers used to quantify biological aging may represent downstream consequences rather than primary mechanisms. In this view, genetic variation and early-life risks could induce epigenetic modifications, which subsequently drive proteomic and metabolic alterations, ultimately manifesting as changes in the clinical biomarkers on which KDM and PhenoAge are constructed [29, 30]. Although limited, there are previous studies that reported associations of early-life exposures with epigenetic aging indicators like GrimAge [31, 32]. Future studies incorporating more comprehensive measures of biological aging, particularly at genetic, epigenetic, and proteomic levels, are still needed.

This study benefits from the large sample size and long follow-up of the UKB, providing strong statistical power. We examined a broad set of early-life exposures, including prenatal, postnatal, and childhood risk factors, applied two established measures of biological age, and used a DAG-guided mediation analysis to enhance the robustness of our findings. However, some limitations need to be addressed. First, early-life exposures were self-reported and collected retrospectively at baseline, which raises the possibility of recall bias and exposure misclassification. Although it is highly unlikely that recall would differ systematically according to future T2D status, misclassification may still occur and is expected to bias the observed associations and mediation effects, most likely towards the null. The effect of misclassification would therefore most likely have resulted in underestimated effect sizes and proportions of mediation. Except for birth weight, exposures were recorded in categories without information on duration or frequency (i.e., breastfeeding and maternal smoking), limiting the effect assessment. We lacked data on some early-life confounders, such as parental socioeconomic status and related family-level circumstances, so residual confounding may remain. Finally, it is widely known that UKB participants do not reflect the general UK population, and our study participants appeared, on average, approximately 10 years younger in biological age than their chronological age. Consequently, both the distribution of early-life risks and incidence of T2D in our analyses were shifted towards a healthier profile, with a lower prevalence of early-life risk factors and a lower incidence of T2D than would be expected in the underlying source population. As a result, the exposure-outcome associations and mediation effects reported in this manuscript are likely to underestimate the corresponding effects in more representative or higher-risk populations. Our results, therefore, should be interpreted with caution, especially when generalizing to the general or high-risk population.

In conclusion, our findings suggest that adverse early-life exposures are associated with accelerated biological aging in adulthood and an increased risk of late-life incident T2D, and that part of the exposure-T2D associations may be mediated by accelerated biological aging. These observational results highlight biological aging as a potential, but not exclusive, pathway linking early-life risk factors to late-life T2D risks and support the rationale for life-course strategies that target early-life and aging-related factors to reduce diabetes risk.

## Supplementary Information

Below is the link to the electronic supplementary material.

ESM 1 (XLSX 838 KB)

## Data Availability

The data of this study are available from the UK Biobank project site, subject to registration and application processes.
